# Evaluation of mechanical properties of three different screws for rapid maxillary expansion

**DOI:** 10.1186/1475-925X-12-128

**Published:** 2013-12-11

**Authors:** Matteo Camporesi, Lorenzo Franchi, Tiziana Doldo, Efisio Defraia

**Affiliations:** 1 Department of Surgery and Translational Medicine, University of Florence, Via del Ponte di Mezzo, 46-48, 50127 Firenze, Italy; 2 Department of Orthodontics, University of Siena, Siena, Italy

**Keywords:** Rapid maxillary expansion, Mechanical properties, *in-vitro* study

## Abstract

**Background:**

The aim of this in vitro study was the evaluation of the mechanical properties the screws for rapid maxillary expansion (RME).

**Methods:**

Three commercially available screws for RME were tested: Leone A2620; Dentaurum Hyrax; Forestadent Palatal Split Screw. All expansion screws were 10 mm in size. For the evaluation of mechanical properties, the screws for RME were adjusted using the same maxillary dental model. An Instron 3365 testing machine with a load cell of 5 kN recorded the forces released by the screws at different amounts of activation (1, 5, 10, 15 and 20 quarters of a turn). Each type of screw was tested 10 times. Comparisons between the forces released by the different types of screws at different amounts of activation were carried out by means of analysis of Kruskal-Wallis test with *post-hoc* test di Tukey (P < 0.05).

**Results:**

The results of this study showed that all 3 expansion devices were able to develop forces that could produce a separation of the palatine processes. The Hyrax and A2620 expanders developed force values over 20 kg and the Palatal Split screws about 16 kg. Both the A2620 and Hyrax expanders showed significantly greater amounts of forces at all the different amounts of activations with respect to the Palatal Split screw.

**Conclusions:**

All tested devices showed the capability of developing expansion forces (16-20 kg) adequate for RME. The A2620 and Hyrax expanders showed a greater level of rigidity than the Palatal Split screw.

## Background

Rapid maxillary expansion has achieved a prominent role in modern orthodontics as a safe, predictable, and effective way to correct maxillary deficiency on the transverse plane in a wide range of clinical conditions [1-6]. From a biological point of view, rapid maxillary expansion (RME) creates large forces at the sutural site over a short period of time and produces immediate midpalatal suture separation by disruption of the sutural connective tissue. Therefore, in a growing subject, RME represents an effective orthopedic therapy [6-10].

Forces produced by this appliance have been reported in the range of 16.6 to 34.8 pounds (7.54 to 15.8 kg) [7]. These heavy forces maximize skeletal separation of the midpalatal suture by overwhelming the suture before any dental movement or physiologic sutural adjustment can occur [6-11].

The clinical management of RME is well established and only few differences characterize the various expansion protocols, such as the number of turns of the midline screw (activation rate of the screw), i.e. rapid or slow expansion, appliance design (banded or bonded acrylic expander), and anchorage on deciduous or permanent teeth [12].

Currently, several protocols have been proposed that allow either to operate a classic rapid expansion or to alternate rapid expansion and rapid constriction to activate the craniofacial sutures [13-15]. The latter activation pattern is particularly effective to enhance the orthopedic effects of postero-anterior traction of the maxilla achieved by a face mask. To accomplish this aim, expansion devices should respond to specific biomechanical needs: the rapid maxillary expander should be as rigid as possible [16].

The literature [17] is very scarce about *in vitro* investigations concerning the forces released by the expansion screw during activation phases. Muchitsch et al. [17] analyzed only the mechanical features of the retention arms of the RME screws. No previous study investigated the mechanical features of all the components (body and arms) of the screws for RME. The purpose of this *in vitro* study, therefore, was to analyze the compression strain developed at each activation of three types of screw for RME (body with arms) utilizing a rigid support.

## Methods

The study used an experimental model reproducing the maxillary dental arch with the palate to adapt all the screws and to standardize the position of the screw body at the same distance from the palate.

**Figure 1:**
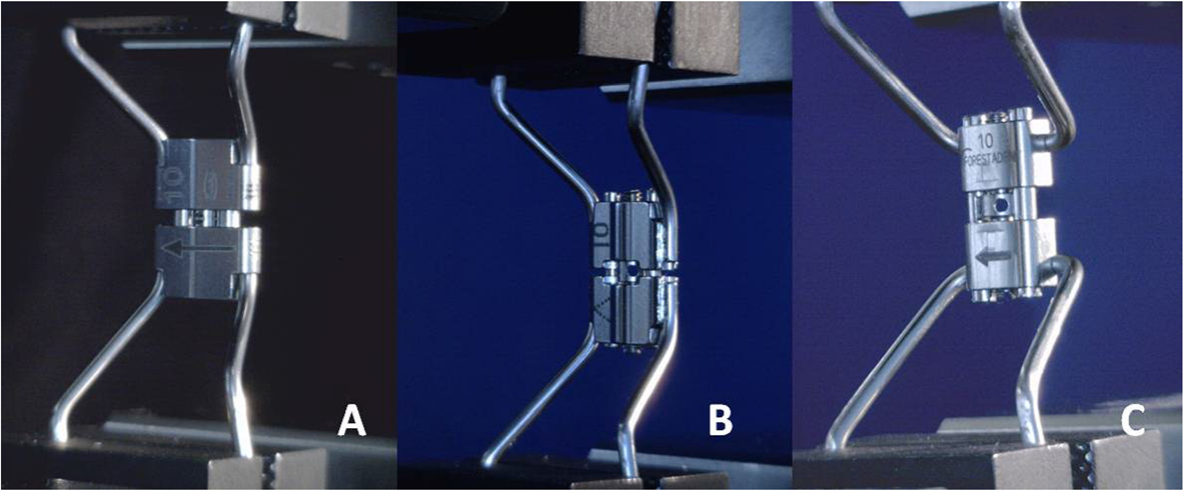
The three types of screws for RME examined: A2620 (A); Hyrax (B); Palatal split screw (C).

We analyzed the stiffness of 3 screws for RME:

•A2620 rapid expander (Leone orthodontic products, Sesto Fiorentino, Firenze, Italy) (Figure 1A);

•Hyrax (Dentaurum, Ispringen, Germany) (Figure 1B);

•Palatal Split Screw (167–1326, Forestadent, Pforzheim, Germany) (Figure 1C).

**Figure 2:**
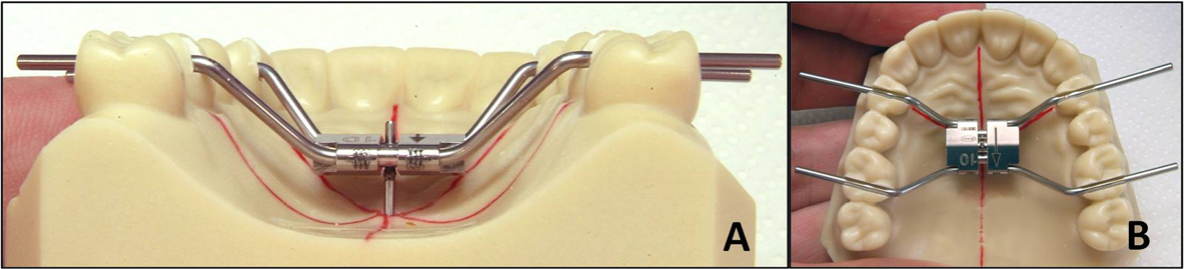
**Demonstrative resin model.** This model reproducing the maxillary dental arch. A pin presenting with a vertical stop at 3.5 mm from the palatal vault was embedded in the resin in the middle of the palate to standardize the position of the screw body **(A)** and of the bends of the screw arms at the level of first premolars and first molars **(B)**.

The expanders presents different technical characteristics: the arms can be either parallel (A2620 and Hyrax) or orthogonal (Palatal Split Screw) to the screw guides. All tested screws were 10 mm in size and a full activation turn was equivalent to an expansion of 0.8 mm (4 activations × 0.2 mm). All tested screws presented the arms laser-welded at the body. In order to evaluate the rigidity of the device, the bending of the arms of the three screws was standardized to replicate a clinical setting (Figure 2). The expansion screws were applied on a demonstrative resin dental model of the upper arch in the permanent dentition, and the arms were bent so that the body of the screw was always at the same distance from the palate. Three red lines were drawn on the resin model (Figure 2A and 2B): one was drawn along the midline while the other two lines crossed the palate from a lingual point at the gingival margin of the first premolar on one side of the arch to a lingual point at the gingival margin of the distolingual cusp of the first molar on the other side of the arch. A metal pin was embedded in the model where the three lines crossed (Figure 2A and 2B). The expansion screw was inserted at the level of the activation hole on the pin that presented with a 3.5 mm stop from the palatal vault. This stop allowed to standardize the position of the expansion screw (Figure 2A). The arms of the expansion screw were bent following the red lines on the palate so that the edges of the arms were placed into grooves carved within the clinical crowns of the first premolars and first molars with a CNC milling machine (Dyna DM2900 three axis milling machine, Sigmatec Precision, Gilroy, Ca, with Bosch Rexroth Indra Control V Computerized Numerical Control, Bosch Rexroth, Lohr am Main, Germany). The grooves connected the lingual and buccal cusps on the first premolars and the distolingual and distobuccal cusps of the first molars (Figure 2A and B). The grooves were 1.5 mm large and 4.0 mm deep at the first premolars and 1.5 mm large and 3.5 mm deep at the first molars. The arms of all 3 expanders had a diameter of 1.5 mm.

**Figure 3:**
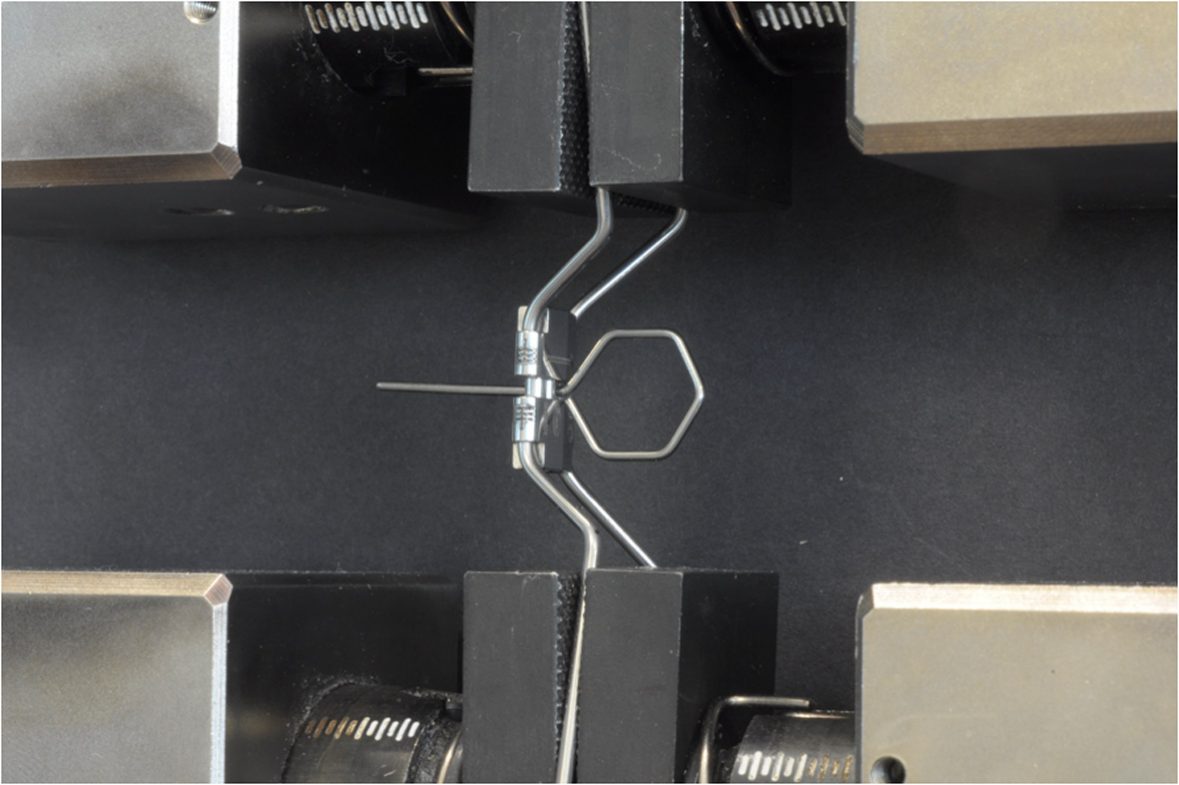
**The specimens were arranged in Instron testing machine.** The expander was placed on the Instron 3365 machine with a load cell of 5 kN by grabbing the arms bent at tooth level with the upper and lower clamps of the machine. The activations were performed by means of a stainless steel (AISI 302) key with a diameter of 1 mm. The key was inserted fully into the holes of the screw.

To estimate the rigidity of the screws, an Instron 3365 test machine was used (Instron Corp, Canton, Ma) with a load cell of 5 kN (Figure 3) to record the forces released by the expander.

The expander was placed in the Instron machine by grabbing the arms bent at tooth level with the upper and lower clamps of the machine, trying to keep the expander as aligned as possible on the vertical plane (Figure 3). Once the device was positioned, the screw was activated one quarter of a turn (i.e. 0.2 mm expansion for all screws) and the resulting compression force was recorded. A total of twenty activations for each expander were carried out. The limit of 20 activations was chosen because deformation of the expansion key occurred in all the three types of expander after this amount of activation (after 9–10 activations for the A2620 and Hyrax expanders). Ten trials for each type of expander were performed, for a total of 30 tests. After each test (20 activations) the expander was replaced by a new one. The activations were performed by means of a stainless steel (AISI 302) key with a diameter of 1 mm. The key was inserted fully into the holes of the screw (Figure 3).

### Statistical analysis

Descriptive statistics of the forces developed by the 3 screws after 1, 5, 10, 15 and 20 activations (each corresponding to one quarter of a turn of the screw) was calculated. Preliminary analysis of the data revealed that normal distribution (Kolmogorov-Smirnov test) and/or equality of variances (Levene’s test) could not be assessed for all the variables. Therefore, statistical comparisons were made by means of nonparametric tests to compare the loading capacity to the different activations within the same expander (Friedman test with Tukey ‘s post-hoc test) and between the 3 different expanders (Kruskal-Wallis test with Tukey’s post-hoc test) (Sigma Stat 3.5, Systat Software Inc., Point Richmond, Ca).

The power of the study for one-way ANOVA was calculated on the basis of the sample size of 10 screws, an alpha level of 0.05, with an effect size 1.5 [18]. The power of the study was 0.83.

## Results

The descriptive statistics and statistical comparisons of the forces generated by the 3 types of expander during the tests are shown in the Table 1.

**Table 1:** Statistical comparison between the different expansion screws at the different numbers of activations (1 activation is equivalent at ¼ of turn)

	**A2620 (1)**			**Hyrax (2)**			**Palatal split screw (3)**			**Statistical comparisons (p<0.05; test di Kruskal-Wallis)**
	**Med.**	**25%**	**75%**	**Med.**	**25%**	**75%**	**Med.**	**25%**	**75%**	
**1 activation (1)**	23.4	18.2	27.4	21.8	6.5	22.7	15.9	14.5	18.0	**1vs3**
**5 activations (5)**	100.3	65.1	122.4	86.6	81.5	109.8	56.6	53.8	61.1	**1vs3; 2vs3**
**10 activations (10)**	170.4	145.9	181.2	150.8	146.3	179.6	103.2	101.7	104.0	**1vs3; 2vs3**
**15 activations (15)**	202.9	196.7	209.3	182.6	179.8	209.6	135.6	126.7	137.7	**1vs3; 2vs3**
**20 activations (20)**	215.0	210.7	217.2	197.9	193.8	221.0	157.8	143.0	159.8	**1vs3; 2vs3**
**Significant statistical comparisons (p<0.05; test di Friedman)**	**1vs10; 1vs15; 1vs20; 5vs15;5vs20; 10vs20**			**1vs10; 1vs15; 1vs20; 5vs15;5vs20; 10vs20**			**1vs10; 1vs15; 1vs20; 5vs15;5vs20; 10vs20**			

The values are expressed in Newtons.

Med.: median; 25%; twenty-fifth percentile; 75%: seventy-fifth percentile.

A total of twenty activations for each expander were carried out. The limit of 20 activations was chosen because deformation of the expansion key occurred in all the three types of expander after this amount of activation (after 9–10 activations for the A2620 and Hyrax expanders). As for the comparisons within the same type of expander (statistical data reported in the columns of the Table 1) the forces developed after 1 activation were significantly higher compared to the forces developed after 10, 15, and 20 activations for all the 3 types of expander. The forces generated after 5 activations were significantly higher than those generated after 15 and 20 activations, for all the 3 types of expander. The forces developed after 10 activations were significantly higher than those developed after 20 activations, for all the 3 types of expander. No significant differences were found between the following amounts of activations for all three expanders: 1vs5, 5vs10, 10vs15, and 15vs20.

With regard to the comparison between the different types of expanders (statistical data reported in the rows of the Table 1) after 1 activation the A2620 expander generated significantly greater forces than the Palatal Split Screw, while there was no significant difference between the A2620 expander vs. the Hyrax and between the Hyrax vs. the Palatal Split Screw. After 5, 10, 15 and 20 activations, the comparisons showed that both the A2620 and the Hyrax expanders generated significantly greater forces than the Palatal Split Screw.

## Discussion

This study analyzed the forces produced by three different palatal standard RME screws imposing the same distances of the body from the palate. The literature [17] is very scarce about *in vitro* investigations concerning the forces released of the screw during activation phases.

A concern about the amount of force of resistance of the maxillary tissues [8] against the RME was the main issue of the papers by Isaacson et al. [6,10]. In these *in vivo* studies, a modified RME was used with a force-measuring dynamometer connecting the expansion screw and the bands on one side of the mouth, while on the other side an acrylic coverage pressed against the palatal alveolar process. The expansion screw opened 0.8 mm per complete turn, thus having a design which is still the most commonly used nowadays. The authors underlined that the decay immediately following an activation was rapid, but that the rate of decay rapidly decreased within several minutes [6,10].

In a more recent study, Halazonetis et al. [19] were able to measure the contribution of the stretched cheeks as resistance to maxillary expansion: it was negligible as it was 0.6 g/cm^2^ per mm of expansion.

In a recent *in vitro* study [17] the authors using a three-point bending test examined only the retention arms of 16 types of RME screws because they assumed that these represented a particularly vulnerable and stressed weak point of RME appliances. The authors found that despite having the same cross-sectional diameter, the single retention arms of all tested stainless steel expansion screws displayed variable loading capacities (force, stress, and deformation parameters) when subjected to three-point bending test. It was stressed also that the relevance of the variation between the tested retention arms lies in the clinical demand. During the pre-pubertal period, the rigidity of retention arms is not as important as in the following periods of increasing interdigitation and ossification of the midpalatal suture [17].

**Figure 4:**
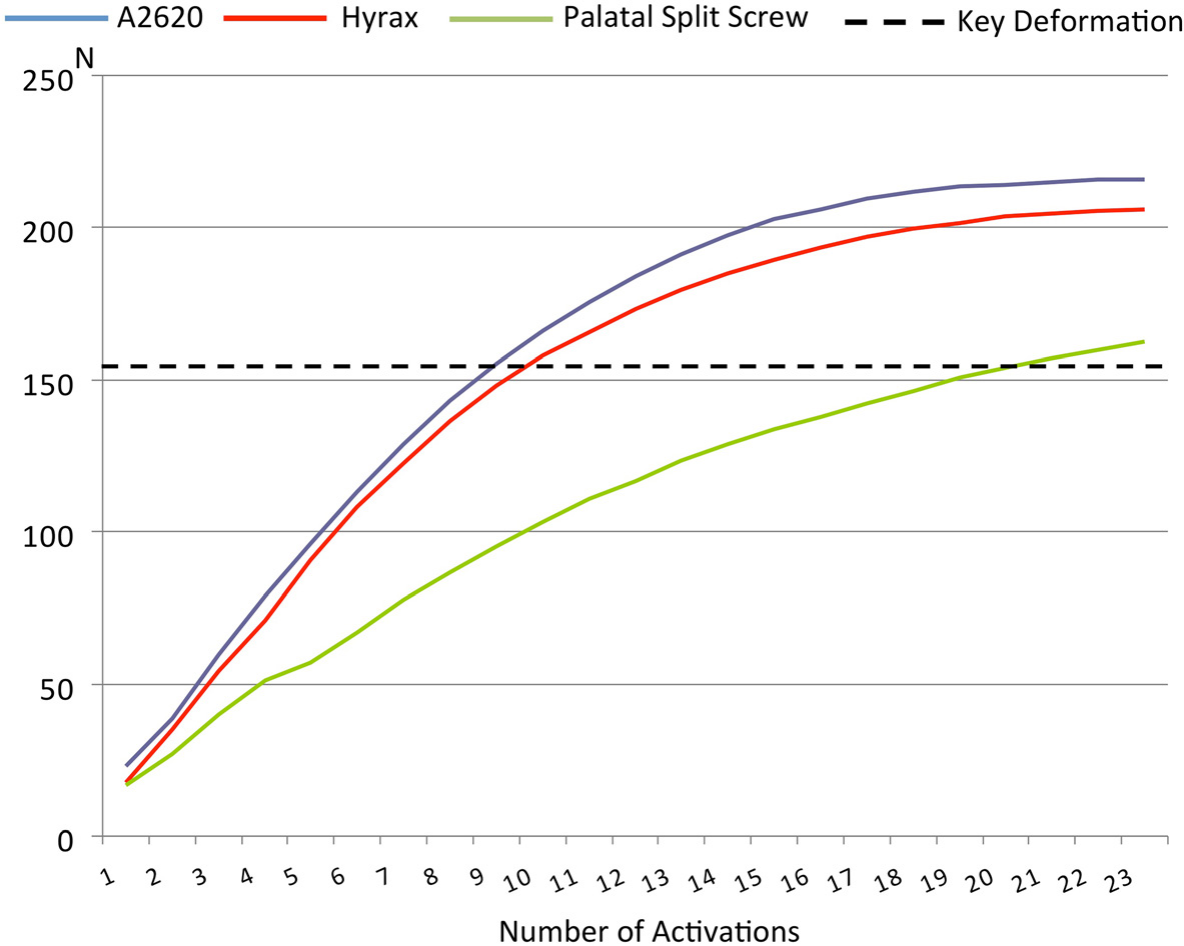
Graphical representation of the screw activation/force curves (forces are expressed in Newtons).

No previous study investigated the mechanical features of the screws (body and arms) for RME. The present study showed that the greater is the stiffness of the expander (see the activation screw/force curve) (Figure 4), the greater is the force developed by an equivalent activation. It should be stressed that the forces registered during the experiment are just forces that the palatal expander can develop and not the forces required to open the midpalatal suture. It’s also obvious that, in a clinical case, the force developed will also depend very much on the rigidity of the midpalatal suture, which is much more resilient than an Instron machine.

The results of this study showed that all 3 devices succeeded in developing enough expansion force to cause a separation of the palatine processes, such as over 20 kg force for both the Hyrax and the A2620 expander and about 16 kg for the Palatal Split Screw. Thus, all expanders showed appropriate clinical skills. In addition there was a very low variation of the values obtained in each group.

The between-group statistical comparison showed a significantly greater stiffness of both the A2620 and the Hyrax expanders, compared to the Palatal Split Screw. Such devices are those that should transfer better the force of the activation of the screw to the teeth and consequently to the bony structures, thus potentially reducing the risk of dentoalveolar tipping.

In all cases the force-activation curve had a similar trend, with an almost linear behavior in the first 10 activations, which decreased and became null at the maximum number of activations. Both the A2620 and the Hyrax expanders showed a trend in the developed force that increased up to about 18–20 activations, after which a “plateau” was reached, while the Palatal Split Screw succeeded in increasing the force up to 24 activations (Figure 4).

**Figure 5:**
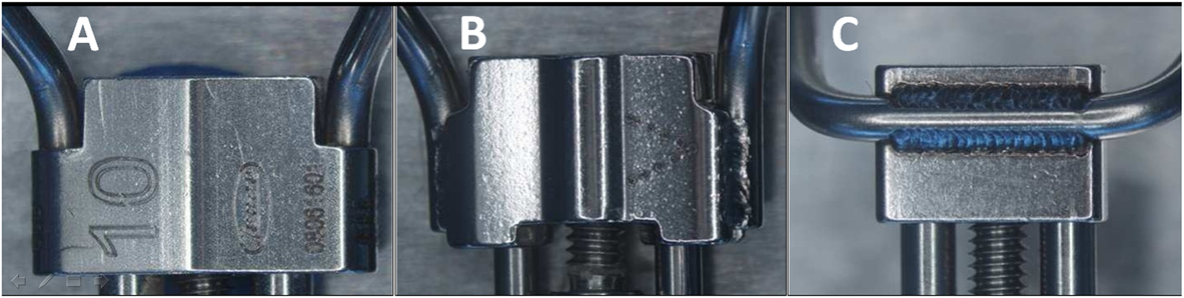
**Technical characteristics of the expanders.** The orientation of the arms to the body of the screw: the arms are parallel to the screw guides on both the A2620 **(A)** and the Hyrax **(B)** expanders, while they are orthogonal to the screw body in the Palatal Split Screw **(C)**, where the greater length of the arms gives less rigidity to the device. All tested screws presented the arms laser-welded at the body.

Both analogies and differences in behavior are can be related to the technical characteristics of the expanders, in particular to the welding of the arms to the body of the screw: they are parallel to the screw guides on the A2620 (Figure 5A) and the Hyrax (Figure 5B) expanders, while they are orthogonal to the screw guides in the Palatal Split Screw (Figure 5C), where the greater length of the arms gives less rigidity to the device. It should be noted that the arm clamping could have introduced additional stiffness. This factor, however, should have affected uniformly the results of the different screws since both the bending and the clamping of the arms was standardized.

All tested expanders showed significant failure of the structure when it came to the twenty-second/twenty-fifth activation, when the developed forces were approximately 22 kg on the A2620 expander, about 20.5 kg on the Hyrax expander, and about 17.5 kg on Palatal Split Screw. It is important to notice, though, that such condition will never occur in a clinical case, since the deformation of the expansion key occurs at about 16 kg.

## Conclusions

The expansion screws analyzed in the current study were able to develop forces of 16–20 kg, which is adequate to obtain a rapid expansion of the maxilla. The expansion devices showed failure of the structure only at high forces (about 23 kg), that cannot be reached in a clinical setting. The A2620 and Hyrax expansion screws showed a greater rigidity compared to the Palatal Split Screw.

## Competing interests

The authors declare that they have no competing interests.

## Authors’ contributions

MC have made substantial contributions to conception experimental design and acquisition of data; LF participated in the design of the study, performed the statistical analysis, and edited the text; TD performed the experiments; ED revised it critically for important intellectual content; and all of the authors read and approved the final version of manuscript.

## Authors’ information

MC Research Fellow, Department of Surgery and Translational Medicine, University of Florence, Florence, Italy.

LF Assistant Professor, Department of Surgery and Translational Medicine, University of Florence, Florence, Italy; Thomas M. Graber Visiting Scholar, Department of Orthodontics and Pediatric Dentistry, School of Dentistry, The University of Michigan, Ann Arbor.

TD Assistant Professor, Department of Orthodontics, University of Siena, Siena, Italy.

ED Associate Professor, Department of Surgery and Translational Medicine, University of Florence, Florence, Italy.
